# Scapular Dyskinesia, the forgotten culprit of shoulder pain and how to rehabilitate

**DOI:** 10.1051/sicotj/2019029

**Published:** 2019-08-20

**Authors:** Andreas Christos Panagiotopoulos, Ian Martyn Crowther

**Affiliations:** 1 Royal Victoria Infirmary Queen Victoria Road Newcastle Upon Tyne NE1 4LP United Kingdom; 2 Northumbria Specialist Emergency Care Hospital Northumbria Way Cramlington, Northumberland NE23 6NZ United Kingdom

**Keywords:** Scapular Dyskinesis, Rehabilitation, Sports injury

## Abstract

The improper movement of the scapula during shoulder movement is termed scapular dyskinesis and is an often-forgotten cause of pain and dysfunction. The scapula is a key part of the upper limb kinematic chain and is a vital component of the glenohumeral rhythm; which is a major determinant of the efficiency and efficacy of the upper limb. We provide an overview of the complex regional anatomy of the shoulder girdle and how this allows the scapula to act as a both a dynamic and static stabilizer to the upper limb. We explore the normal biomechanics and the aetiology, epidemiology and pathological occurrences which can disrupt the normal function and lead to scapula dyskinesis. Scapula dyskinesis is a poorly understood condition and provides a challenge for the clinician in both diagnosis and management. We provide a summary of the clinical assessment which is most likely to identify the source of the pathology and guides the treatment which is largely rehabilitation of the musculature with focused and specialized physiotherapy.

## Introduction

The glenohumeral joint (GHJ) is the gateway between the axial skeleton and the upper limb. The glenoid fossa and the humeral head operate in a complex synergistic fashion to permit the multiplanar movements of the joint. The balance between joint stability and freedom of movement is regulated by static (bony shapes, ligaments) and dynamic (muscles) factors. The GHJ is a rather unstable joint compared to the other ball and socket joints in the body, but the aforementioned factors provide relative stability in multiple planes of motion. In disease, the majority of patients complain of loss of function and pain, with the rotator cuff, shoulder capsule and impingement being the most common culprits. In contrast disorders of the scapula are often neglected due to a lack of awareness and expertise in assessment. This review highlights *scapula dyskinesis*, “the abnormal anatomy and kinetics of the scapular” and with the aim to (a) improve the understanding of the biomechanical principles of the scapular function, (b) study related pathophysiology in different disease processes and (c) delineate the rehabilitation regimes available for the management of the disease.

### Scapula anatomy

The scapula is a complex triangular bone on the posterior thoracic cage between the levels of T2 and T7. It is composed of:

- The Anterior (costal) surface – Has a concave surface which serves as attachment for subscapularis and serratus anterior. The Coracoid process originates from the superior lateral anterior surface. This is a “finger-like” projection where Pectoralis Minor, Biceps Brachi (Short Head) and Coracobrachialis attach. At the superior aspect of the anterior surface is the attachment of Omohyoid, one of the strap muscles [1].
- The Lateral surface – Contains the glenoid fossa, the scapula portion of the glenohumeral joint. Also located here are the Supraglenoid and Infraglenoid tubercles which provide attachment for Long head of Biceps Brachii and Triceps Brachii, respectively [1].
- The Posterior surface – Contains the boney structures of the spine, acromion, supraspinous fossa and infraspinous fossa. The spine and acromion contain the attachment of trapezius and deltoid, whereas the supraspinous and infraspinous fossae serve as attachments for supraspinatus and infraspinatus, respectively. The inferior lateral posterior surface also provides attachment for Teres Minor, Teres Major and Latissimus Dorsi [1].

The Medial Surface – Provides attachments for Levator Scapulae, Rhomboid Minor and Rhomboid Major [1].

In addition to the various muscle attachments there are two articulating joints. First is the acromioclavicular joint, supported by the Trapezoid and Conoid ligaments attaching to the coracoid process and the acromioclavicular joint capsule which incorporates the acromioclavicular ligament. The clavicle serves three roles:

- Supports the arm, keeping the humerus away from the thorax;
- Protects the cervicoaxillary canal;
- Acts as a means of force transfer from the core to the arm [1].

The second joint is the glenohumeral joint which is stabilized by four anterior ligaments, the superior, middle and inferior glenohumeral ligaments and the coracohumeral ligament. Posterior stability is aided by the posterior capsule.

In addition to the articulating joints, there is the articulation between the scapula and thorax to consider. Although no boney articulation occurs here, it is allowing a vast degree of “gliding” movement in a 3-dimensional plane. The role of the scapular and its muscular attachments is to dynamically control the position of the glenoid to allow optimal biomechanical movement at the glenohumeral movement joint.

### Scapular biomechanics

The scapula serves four biomechanical roles:

- It is the centre of rotation of the humerus.
- It is the anchor of the humerus onto the thoracic wall.
- It keeps the acromion from obstructing the movement of the humerus both in abduction and in flexion, thus there is no impingement.
- It is the means by which forces are transmitted from the core to the arm.

Given the scapula’s integral part of the upper arm’s kinematic chain, the scapular position and thus the glenoid position dictate the degrees of freedom within each plane of shoulder movement [2].

**Figure 1:**
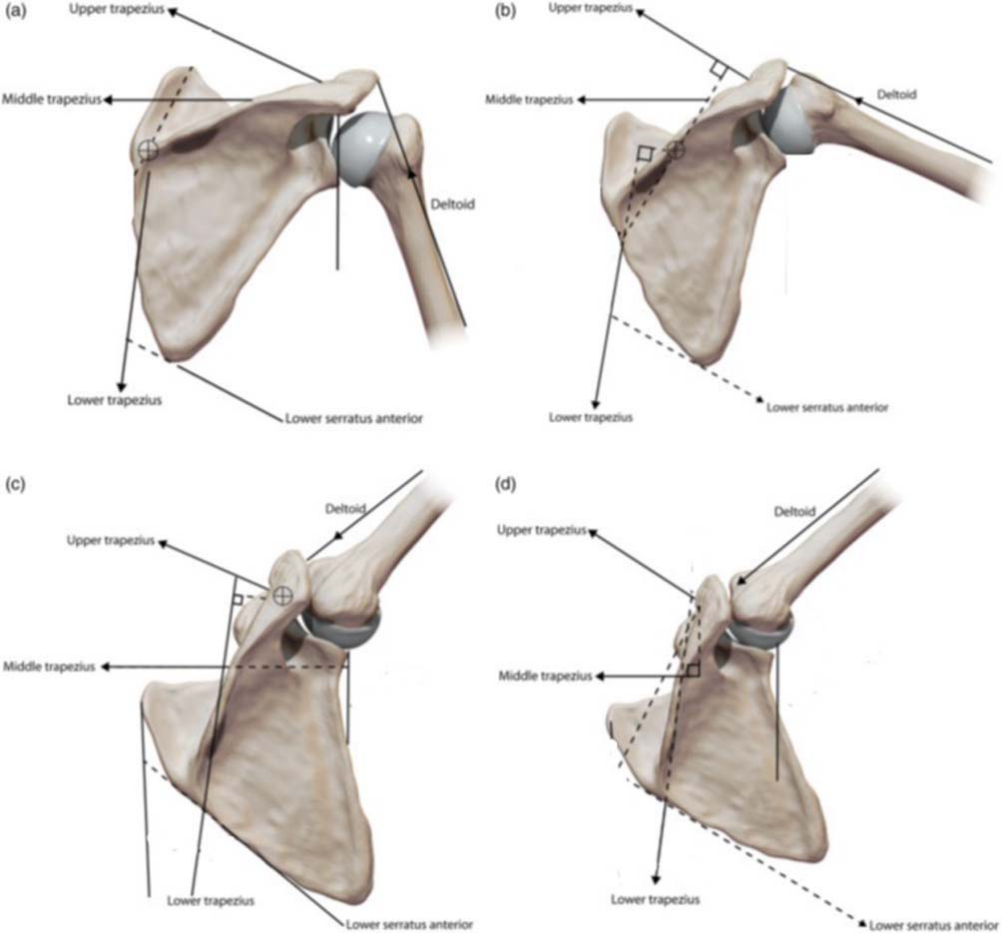
The scapular movement in relation to humeral abduction and the corresponding muscle vectors that affect it.

To permit this, the scapula can move in the following ways (Figure 1):

- Elevation/depression;
- Protraction/retraction;
- Internal/external rotation;
- Superior/inferior rotation;
- Anterior/posterior tilt.

Analysis of the key shoulder movements of the shoulder, flexion and abduction has provided a comprehensive appreciation of the movement stages involved. It is understood that for these movements to occur, the glenohumural joint and the scapulothoracic articulation move in harmony. Inman et al. found that for the first 30 degrees of flexion and 60 degrees of abduction of the humerus, the scapula seeks to find a position of stability to optimize the power of these movements [3]. In some cases, either the scapula would remain fixed with the glenohumeral joint being the principle area of movement or the scapula would translate medially or laterally to assist the glenohumeral motion. The study concluded that for early degrees of movement, the movement of the scapula was person-specific, with variation seen [3]. The optimal position that the scapula found was termed the setting phase. Once flexion or abduction exceeded those levels, the behaviour of the scapula was much more uniform, with a ratio of movement between glenohumeral and scapulothoracic angle of 2:1, for instance for 15 degrees extension of the humerus, 10 degrees would occur at the glenohumeral joint, 5 degrees at the scapulothoracic.

More recent studies have suggested a less variable pattern of scapular movement, with the key component being upward rotation, followed by posterior tilt and external rotation. Research has highlighted that the upper and lower trapezius along with serratus anterior are the muscles that mostly affect scapular movement and cause dykinesia. When the scapula biomechanics are considered in relation to the anatomy, it becomes evident that the combination of movements, planes and muscles involved there is a vast array of combinations that could lead to abnormal movement function [3–5].

### Scapular pathophysiology/pathomechanics

The causes of scapular dyskinesis can be split into three groups:

- Shoulder-related;
- Neck-related;
- Posture-related [6].

a) *Shoulder-related causes of scapular dykinesia*

Shoulder related – The shoulder pathologies are the most common origin of complaints. Almost all shoulder pathologies are accompanied with a degree of dyskinesis . The most common pathologies that are associated with some form of scapular dyskinesis are: (1) acromioclavicular instability, (2) shoulder impingement, (3) rotator cuff injuries, (4) glenoid labrum injuries, (5) clavicle fracture and (6) nerve-related. The common characteristic of all these pathologies is the disturbance of the scapulohumeral rhythm [7, 8].

Shoulder impingement is associated with greater scapular protraction (in the resting positions), greater posterior tilt (during abduction) and greater internal rotation (during plane elevation). Furthermore, the scapula shows less upward rotation when the scapular plane is elevated [9].

The scapula has a different performance pattern in shoulder instability, with reduced rotation when the arm is elevated, but increased internal rotation when the scapular plane is raised [10].

In frozen shoulder, the scapula externally rotates earlier and at a greater degree compared to a normal scapula. But research has failed to show that the increased mobility of the scapula is a compensatory mechanism [10, 11].

As mentioned earlier in the Biomechanics section, the scapulohumeral rhythm can be disturbed either by inappropriate pattern of muscle activation (too slow or too fast) or inappropriate force of muscle contraction (too strong or too weak). Many muscles acting in different directions influence the scapula, and it is understandable that the timing and force of muscle activity dictates its movement [12, 13].

Fatigue is an important determinant of muscle performance. McQuade et al. have shown that with increasing fatigue the scapulohumeral rhythm is less effective. It would be interesting if the same experimental setup were extended to more complex activities, including more muscles. That way researchers could observe 1) muscle fatigue following real-life movements, 2) which muscles were more susceptible to fatigue and 3) if muscles assume dominance once the synergists are fatigued [2]. Other muscle problems, such as stiffness of the latissimus dorsi, have been reported to affect the rotation of the scapula, pulling the bone superiorly [14].

The trapezius and the serratus anterior muscles have been linked to the development of dyskinesis in both shoulder impingement and shoulder instability. In impingement, the upper and lower trapezius along with the serratus anterior have altered their activation pattern, with the trapeziae showing a greater strength of activation compared to the serratus anterior [15].

Rotator cuff arthropathy promotes the increased action from the rotator cuff muscles, supraspinatus and infraspinatus, and from the upper trapezius when compared to symptomatic patients [16].

The soft tissues that surround the shoulder have been linked to the development of altered scapular mechanics. Namely, both pectoral muscles (major and minor) and the glenohumeral capsule have been identified as important factors. The tightness of the muscles of the pectoral region promotes anterior translation of the shoulder girdle and consequently the scapula [17]. Furthermore, stiffness of the posterior aspect of the glenohumeral capsule shows an altered resting scapular position, further anteriorly compared to normal individuals, a similar pattern to shoulder impingement [18].

b) *Neck-related*

There are two subtypes of neck pathologies that can affect the shoulder: 1) “mechanical neck pain” syndromes and 2) cervical nerve root-related syndromes. “Mechanical neck pain” syndromes are defined as a group of pathologies affecting the joints (degenerative changes) and muscles (e.g. fatigue or imbalance) of the neck. It has not yet been established how the symptoms get referred to the shoulder, but one can appreciate the proximity of such structures to the area. It has been postulated that body posture affects muscle strength. In fact, because of the western style of living and the extensive use of computers, patients acquire a “slouched” posture. As a result, the cervical and upper thoracic spines lose their naturally occurring curvatures [8].

Conversely, the link between *nerve pathologies* (e.g. nerve root compression or avulsion) at the neck and shoulder-related complaints is well established. All the nerves that provide sensory and motor supply to the shoulder originate from the brachial plexus, especially from the C5 and C6 roots, and the accessory nerve (it transverses from the upper portions of the spinal cord and the lower parts of the brain towards the sternocleidomastoid muscle) [7]. Pathologies arise when the nerves inappropriately activate one or more nerves around the scapula and consequently disorganize the rhythm of scapular movements relative to the main skeleton or the upper limb. The pattern of muscle activation is an important part of clinical assessment and rehab as explained later.

c) *Posture-related causes of scapular dyskinesis*

Excessive thoracic kyphosis and cervical lordosis alter the resting position of the scapula. Athletes are more susceptible to these changes. Depending on their sport, they develop core muscle imbalances that alter spinal curvatures and soft tissue tensions [13].

*Epidemiology of scapula dyskinesis*

The shoulder joint plays an important role in the function of the upper limb and in the activities of daily living. Shoulder pathologies are very common with the lifetime risk being between 40% and 60% [19]. In particular, athletes that principally use their arm over their head (e.g. volleyball, handball, swimming, tennis) are at a higher risk of injuring one of the structures of the shoulder [19, 20]. The other high-risk group are individuals who use personal computers [21].

Scapular dyskinesis has been detected in individuals with or without symptoms. It is closely linked with shoulder instability and shoulder impingement syndrome [18].

### Clinical assessment

The clinical assessment of the scapula is divided into three stages: (1) Direct observation; (2) Manually Assisted Movements and (3) Assessment of surrounding structures [22, 23].

To perform *Direct observation of the scapula* the patient’s resting scapular position is assessed followed by the observation of active movements; stands and holding a 1-kg bag and is asked to perform simple active movement; shoulder flexion and abduction, whilst the examiner observes for winging, early elevation, rapid downward rotation and shoulder shrugging. The findings are noted as a yes/no answer, followed with a description of the best performance [8, 12].

*Manually assisted movements of scapula*: two tests are involved in this step, the scapular assistance test (SAT) and the scapular reposition (retraction) test (SRT). The SAT involves the examiner pushing the inferior-medial border of the scapula outwards and upwards whilst stabilizing the upper medial border when the patient has his humerus elevated. This test assesses how different the pain is perceived. In a positive test the pain is reduced and it is usually positive in patients with painful arc or shoulder impingement.

**Figure 2:**
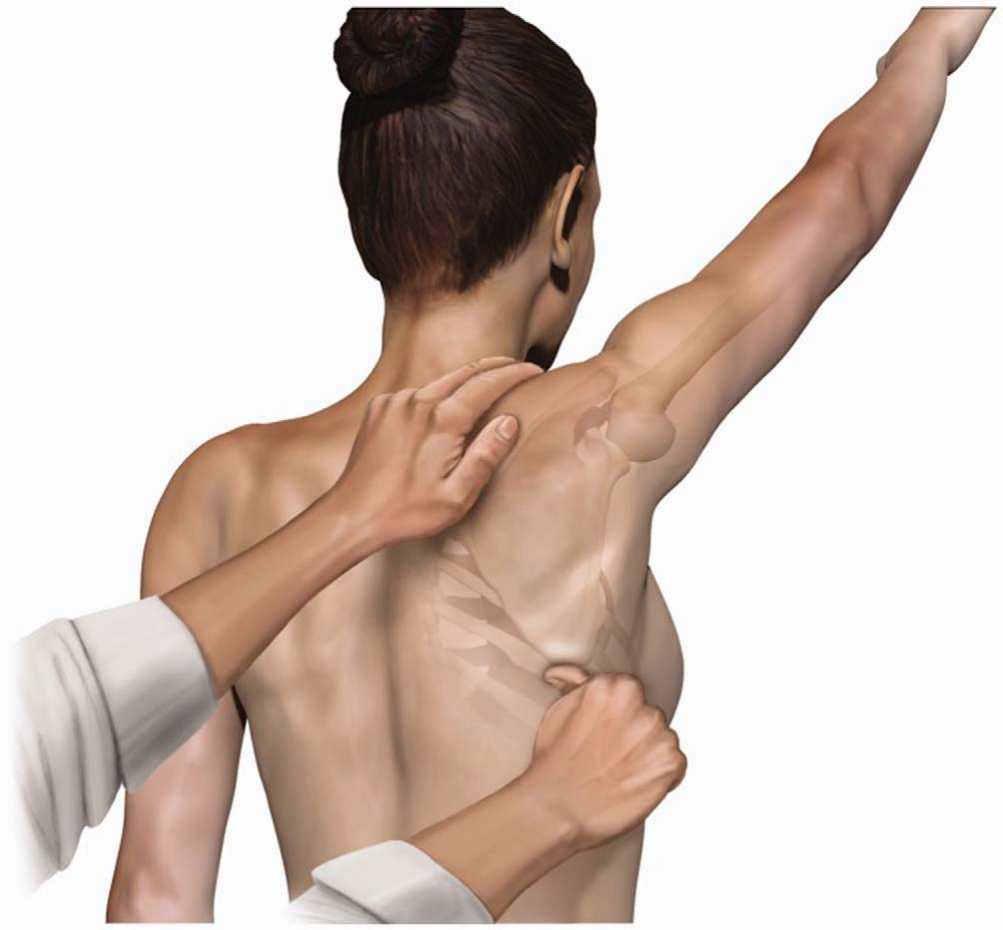
The scapular assistance test (SAT), a manually assisted examination manoeuvre.

**Figure 3:**
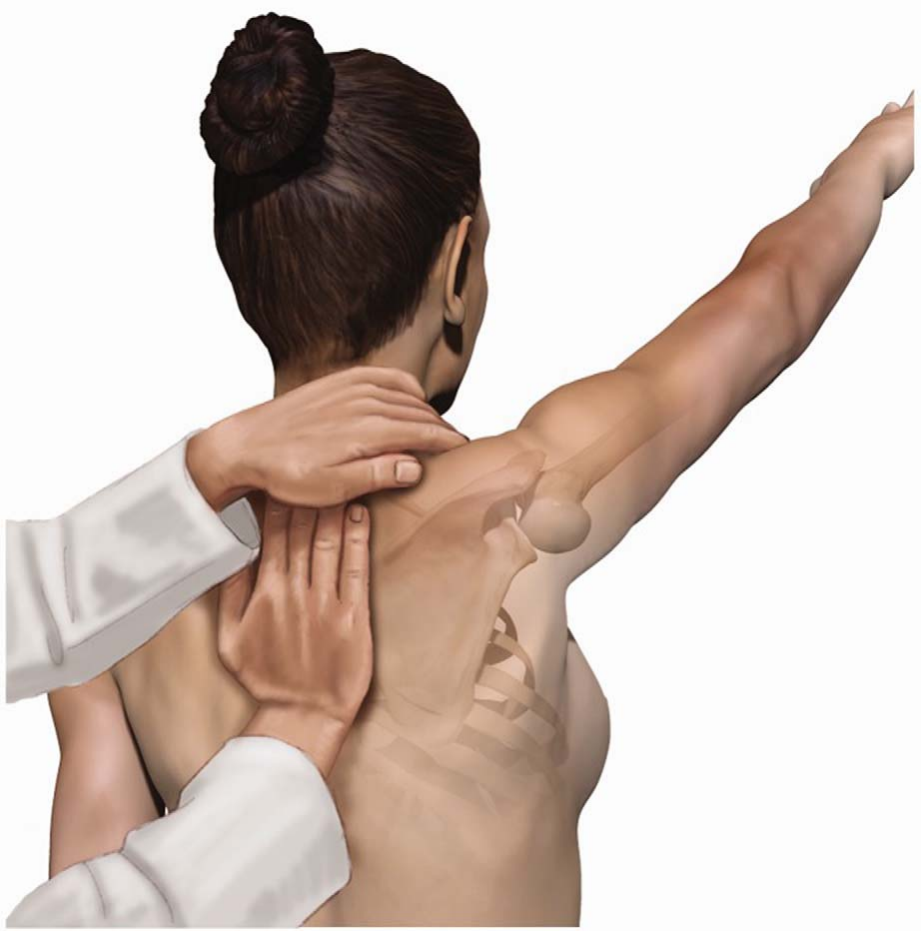
The scapular reposition (retraction) test (SRT) a manually assisted examination manoeuvre.

There are no false positives in asymptomatic patients (Figure 2) [22–24]. In SRT the examiner has to position and stabilize the medial scapular border with one hand, whilst the patient is asked to elevate his arm isometrically (no change in the angle of the joint) against the examiner’s other hand. Again the test is positive when this manoeuvre reduces the pain felt by the patient. This test is also positive if the patient’s strength is increased during the isometric elevation of the arm. The scapular reposition test is sufficiently specific and sensitive in rotator cuff injuries (Figure 3) [8].

3) *Assessment of surrounding structures*: the structures around the scapula (thoracic spine, the acromioclavicular joint, rotator cuff muscles, two heads of the biceps and the glenoid labrum) are assessed. It is important to assess these structures thoroughly in order to exclude or confirm alternative causes of the symptoms. The assessor is looking for symptoms (pain, loss of function) in other structures, soft tissue laxity and muscle power [8].

## Treatment of scapular dyskinesis

Scapular rehabilitation should be part of a broader programme of shoulder physiotherapy to address the functional needs of the individual patient and the concurrent deficiencies of neighbouring structures, such as the shoulder or the neck. Physiotherapy can be either an adjunct to surgical repair of structural injuries or a standalone approach to the management of the patient’s symptoms. The main goal of therapy is to improve the kinematic chain at different levels from the cervical and thoracic spine to the shoulder. The clinical assessment should identify if scapular dyskinesis is a deficit in soft tissue mobility or muscle action.

Deficits in flexibility include different muscle groups and joint components. The mainstay treatment is stretching of the affected structure to increase the working length. The pectoralis muscle is best stretched by the technique “unilateral corner stretch”, a technique that involves the passive abduction of the humerus at 90 degrees from the resting position [25].

**Figure 4:**
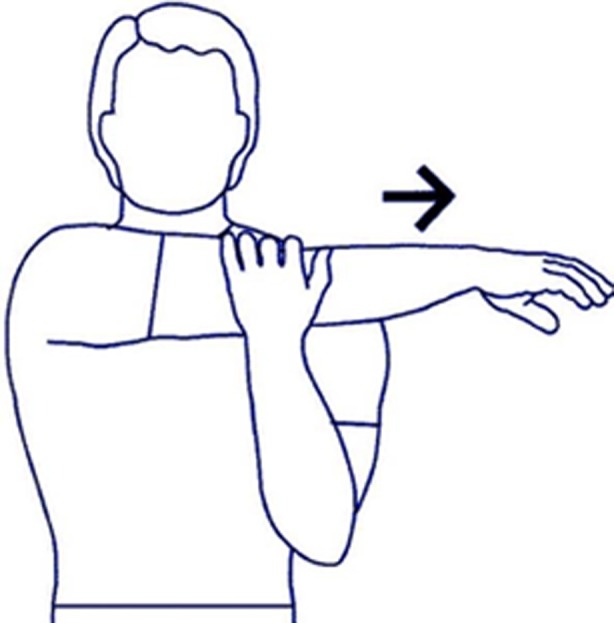
The “cross body stretch”, a useful technique to relax the posterior capsule of the glenohumeral joint.

The posterior capsule of the glenohumeral joint best responds to techniques such as “sleep stretch” and “cross body stretch” which improve the mobility of the joint (Figure 4) [26].

### Rehabilitation of musculature

The rehabilitation of muscle activation patterns is split into three stages: (1) “active conscious control”, (2) “strength and control for daily activities” and (3) “control in athletic performance”. The muscles involved are the serratous anterior and the three parts of the trapezius (superior, middle, inferior) [27]. The average prescribed duration of such programmes is 12 weeks with satisfactory functional outcomes [28]. Specific groups that have higher needs such as volleyball players should undergo longer programmes, around 3 months [29].

1. *Active conscious control*

The scapular musculature requires re-orientation in order to re-engage the correct pattern of activation. The inferior part of the trapezius can be orientated with “scapular orientation exercise” that promotes targeted re-engagement of the muscle under tactile feedback from the other limb [30]. Research has shown that conscious training of the muscles has definite improvements in the kinematic chain but the results can be reversed [31].

Further to the rehabilitation of the muscles, the surrounding structures need to be involved. Especially, the resting position of the spine needs to be addressed. The patient is taught how to maintain a neutral spinal position, respecting the curvatures of the spine at the different levels. This retraining begins from the lumbar spine, followed by the thoracic and finally the cervical spine. The effect is to re-engage the paraspinal stabilizing muscles to maintain a neutral spinal position. It is advised the patients practice this activity multiple times throughout the day [32].

*2. Strength and control for daily activities*

**Figure 5:**
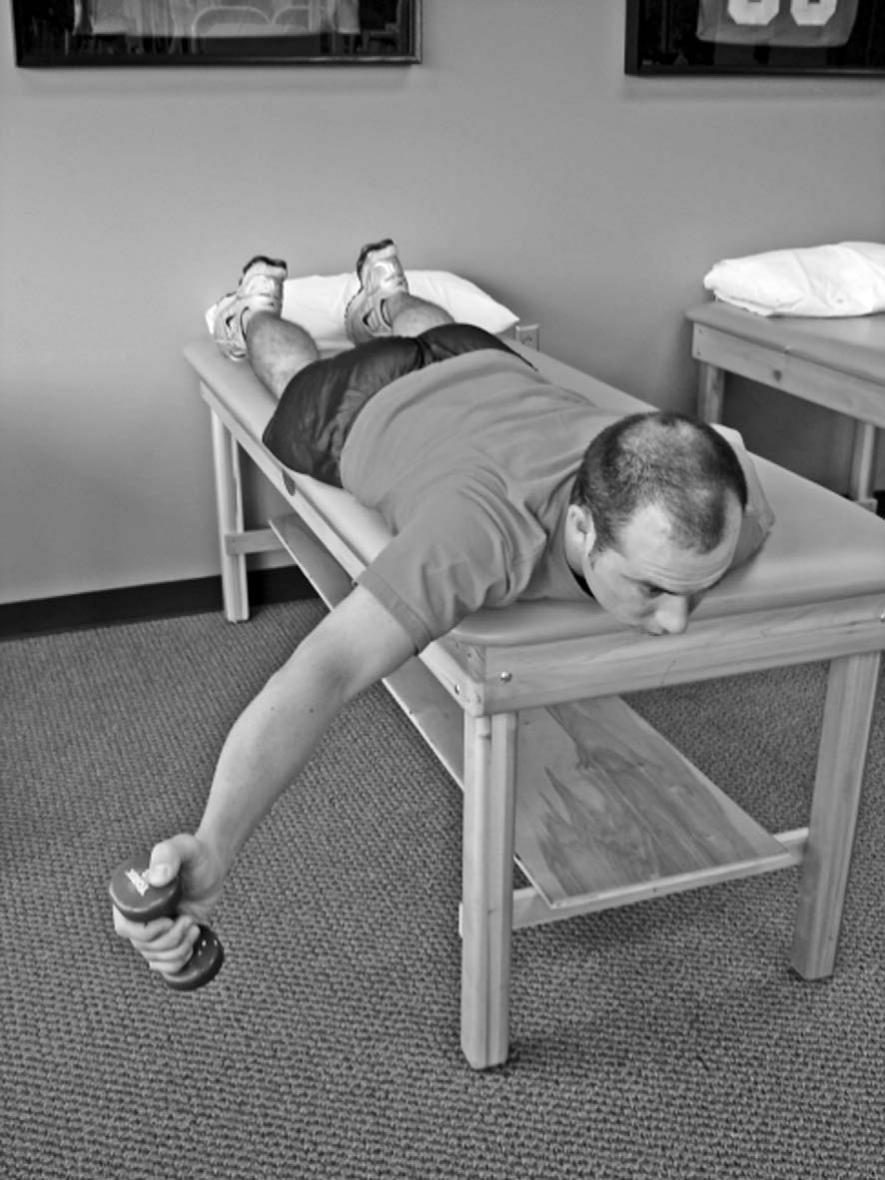
An example of open chain exercise that promotes engagement of the rhomboid and the supraspinatus.

The main concept of this stage is concurrent activation of muscles in order to perform activities of daily life. The prescription should include both “open-chain” and “closed-chain” activities. The exercises should be repeated under different weight bearing conditions. “Open-Chain” activities include “low row”, “inferior glide”, “lawnmower” and “robbery” exercises, that re-engaged the rhomboid muscle (Figure 5). “Closed-Chain” activities aim to promote the awareness of the joint in space (proprioception) and coordination of the rotator cuff muscles [33]. Moreover, muscle strength can be achieved by engaging the deficient muscles in isolation whilst minimizing the activity of the stronger ones [34].

*3. Control in athletic performance*

Depending on the sport and the functional needs of the individual, a detailed prescription of muscle strengthening exercises should adhere to the principles of “scapular control” and “task specific muscle strength” [35].

## Conclusion

The scapula is an under-appreciated component of the shoulder kinematic chain. The importance is highlighted by the significant improvements in functional ability after rehabilitation.

Clinical evaluation of the scapular resting position and function is paramount for the prescription of the necessary physical therapy exercises.

## Conflicts of interest

ACP and IMC have no conflicts of interest to declare.
